# Sexual and Reproductive Health: Knowledge, Attitude, and Perceptions among Young Unmarried Male Residents of Delhi

**DOI:** 10.1155/2015/431460

**Published:** 2015-08-06

**Authors:** Jitendra Kumar Meena, Anjana Verma, Jugal Kishore, Gopal Krishan Ingle

**Affiliations:** ^1^Department of Community Medicine, Maulana Azad Medical College, New Delhi 110002, India; ^2^Department of Community Medicine, Vardhman Mahavir Medical College, New Delhi 110029, India

## Abstract

*Context*. Men play a significant role in all spheres of domestic life including reproduction. Youth is a period of critical development and ignoring sexual and reproductive health (SRH) needs of young men ought to have wider social and health consequences. *Aims and Objectives*. To assess the knowledge, attitude, and perceptions regarding SRH among young unmarried men (18–25 years). *Settings and Design*. A semiqualitative study conducted across four health centers (2 rural, 2 urban) across Delhi. *Materials and Methods*. Focus group discussions (FGDs) were held among sixty-four participants regarding various aspects of SRH. *Data Analysis*. The data generated were analyzed using free listing and thematic content analysis along with simple quantitative proportions for different variable groups. *Results*. Good knowledge regarding HIV/AIDS was observed though found poor regarding other STIs/RTIs. Inadequate knowledge and negative attitude towards SRH and condom use were observed among rural participants. Peer group and mass media were the commonest SRH information sources among rural and urban participants, respectively. *Conclusions*. Poor SRH knowledge, perceptions, and available nonformal, unreliable information sources expose young men to poor SRH outcomes. Early, comprehensive SRH information provision can have life-long protective benefits to them and their partners.

## 1. Introduction

Good reproductive health should include freedom from risk of sexually transmitted diseases, the right to regulate one's own fertility with full knowledge of contraceptive choices, and the ability to control sexuality without being discriminated against because of age, marital status, income, or similar considerations [1]. Male involvement in reproductive health issues had been poor in India and women often depend on husband and other family members for making decision in sexual and reproductive health matters [2]. Men are mutually indispensable partners in sexual and reproductive relationships, marriage, and family building and should be potential partners and advocate for good reproductive health rather than bystanders, barriers, or adversaries. The WHO (1998) emphasized that men should be empowered through the provision of information and services targeting boys, youth, and adults within home, community, and work settings.

With advent of HIV/AIDS pandemic, increasing prevalence of STIs, and the problem of unwanted pregnancies, need of male involvement has become important than ever before [3]. A study done in Bangladesh found significant association between husbands' fertility preferences, background and socioeconomic characteristics, and current use of any family planning method by the couple [4]. The problem is profound in rural areas where inadequate health services, lack of information, social stigma, and policy barriers combined with personal and cultural fears predispose young people to poor knowledge, attitude, and practices regarding SRH. In a study conducted on young unmarried rural Indian men it was seen that their sexual and reproductive health (SRH) knowledge is limited, although the majority were familiar with condoms (99%). Electronic mass media (67%) were the prime source of reproductive health information, yet they lacked detailed knowledge of various contraceptives and felt ignored by health providers, who, they felt, would be capable of providing SRH information through interpersonal communication [5].

A similar study among Khairwar tribe males reported that only 17% of respondents had ever heard of HIV/AIDS infection and STI/RTIs and most had no proper knowledge of its transmission [6]. Lack of correct knowledge regarding reproductive and sexual health among men could be detrimental to their SRH and of their sexual partners. Early sexual activity outside marriage exposes many young men to the risks of unwanted pregnancy and STI/RTIs. In a study done in Turkey, male university students who were sexually active lacked enough knowledge about family planning methods and were reported to engage in high risk behavior [7]. In a similar study in Mumbai, India, it was reported that most sexually active college students did not use condoms and a substantial number of them had sex with commercial sex workers (CSW) [8].

Young adulthood is a period of critical development involving physical, physiological, cognitive, and psychosocial changes. Increasing evidence exists that ignoring SRH needs of young men has wider social and health consequences. In a study done in two boys and two girls' senior secondary schools of rural Delhi, it was found that 25% students had premarital sexual experience [9]. In a study done among male college students of Mumbai it was observed that exposure to erotic materials, a liberal attitude towards sex combined with poor knowledge, and perceived peer norms regarding sexual behavior are some of the most important predictors of risky sexual behavior among male students [10].

Due to the sensitive nature of the subject, little is known about burden and factors associated with SRH problems among men and their knowledge, attitudes, and practices. A study carried out on 120 men attending a reproductive health checkup in a village in rural West Bengal, India, found that SRH issues prevalent and concerning men were sexual weakness, itching around genital areas, burning sensation during urination, early ejaculation, wounds on the genitals, white discharge, and so forth. Other issues raised by men included masturbation, nocturnal emission, consequences of loss of semen, menstruation, pregnancy, and AIDS [11]. Despite wide range of morbidities concerning SRH, treatment seeking behavior among people remains poor as shown in surveys conducted in Bangladesh and India where most people with STI symptoms seek care from unregulated (untrained) private practitioners. The National AIDS Control Organization in India estimates that only 5–10 percent of patients with STIs present to public sector health care. This is true not just for STIs, but for a wide range of curative services, and it is not only the economically wealthy who seek private medical care; the poor also choose private providers for a variety of reasons [12].

Young men are frequently more willing to consider alternative views about their roles in reproductive health and if brought into a wide range of reproductive health services, better SRH outcomes can be expected however; despite all the evidence, there remains a poor male involvement in SRH matters and keeping in view such deficiency the current study was planned and conducted.

## 2. Subjects and Methods

### 2.1. Study Locations

The present study was carried out at four different locations (health centers) affiliated to the Department of Community Medicine, Azad Medical College, New Delhi. These health centers were conveniently chosen as they were functioning under investigator's parent department and had well-maintained population records to facilitate community health action. Two of the health centers were located in rural areas (Pooth and Barwala village) and the other two were urban health centers (Balmiki basti slums and Gokulpuri resettlement colony).

### 2.2. Study Design

It was a semiqualitative study, conducted to assess young men's SRH information needs, perceptions. and preferences through focus group discussions (FGDs). Semiqualitative research involves systematic integration, or “mixing,” of quantitative estimates within qualitative data therefore permitting a more complete understanding and synergistic utilization of data.

### 2.3. Sample Design and Implementation

A convenient sample size of sixty-four was predetermined by the authors based on availability of resources and time constraints. Therefore, to maintain uniformity sixteen participants were included from each health center by selecting first eight eligible men attending general outpatient clinic on two randomly selected days. In case of any refusal the next eligible person was asked to participate in the study. Nonpermanent residents, those not falling in age bracket (18–25 years), and participants with SRH complaints were excluded from the study.

### 2.4. Study Methodology

Selected participants were informed regarding the subject and objectives of the study and informed verbal consent was obtained. The majority of participants were participating for the first time in similar discussion. Prior approval for conducting the study was taken from designated officials at the health centers and community members. A total of eight FGDs consisting of eight participants in each session were held at selected health centres. The study participants were grouped on the basis of their place of residence (rural/urban) during data collection. However, during data analysis stage they were grouped on the basis of residence, education, and age for differential comparisons. The FGDs were carried out at designated health facilities in separate rooms with adequate facilities and privacy. Due to confidentiality of the study participants proper care was taken during data analysis and publication. Study participants were not paid any incentives for participating in the study.

Discussions were held in Hindi language which is the vernacular language of the region and was easily understood by participants. With participants' permission, audio tapes were used to record proceedings from the FGDs. Two male researchers, one investigator and the other recorder/facilitator, conducted the sessions ranging in length from one to one and half hours. To facilitate equal participation, ground rules were communicated to the participants at the outset of discussions. Group members were asked to respect other participants' confidentiality and privacy, wait for their turn to sign or speak, and respect the right of everyone to freely express their views.

Sexual and reproductive health (SRH) is defined as “a state of complete physical, mental and social well-being and not merely the absence of disease or infirmity, in all matters relating to the sexuality and reproductive system. Sexual and Reproductive health therefore implies that people are able to have a satisfying and safe sex life and that they have the capability to reproduce and the freedom to decide if, when and how often to do so” [13]. Good SRH knowledge indicates sufficient knowledge of key sexual and reproductive health (SRH) topics and issues. The topics should reflect those of primary importance for protecting and promoting SRH. In the present study participants were asked to express their views on similar topics such as SRH and its importance, knowledge regarding various SRH problems, prevention, and treatment of reproductive diseases, and high risk behavior; SRH problems and issues refer to multiple disorders which afflict sexual and reproductive functions in men, for example, testicular swelling, loss of libido, urinary symptoms, erectile difficulty, RTI/STI, HIV, infertility, and genital cancers.

A structured vignette about two fictional people from either gender was used to contextualize and simplify the discussions and to provide an acceptable means of exploring potentially sensitive issues when needed. During each focus group session, the investigator led the discussions while the recorder summarized and noted down important points that were made for each of the questions which were used to back up the audio recordings. After each session, the participants were provided with basic SRH information package based on the feedback received.

### 2.5. Data Analysis

The data collected in form of audio recordings and notes was translated into English by three authors and after cross-checking a consensus final report was made. Transcripted data were organized into categories and content thematic approach was used for analyzing data using categories from the dataset. Relevant quotes were also mentioned in the text to illustrate these categories. Simple quantitative proportions for different variable groups were also used to analyze the data.

## 3. Results

A total of seventy-eight people were offered participation in the study with a response rate of 82.1% (*n* = 64). The median age of participants was 23 years and half of them were residing in rural areas of Delhi. The majority of participants had completed high school education (10th std.) and almost half of them were pursuing higher education at the time of data collection. The following themes were identified in the data: (a) knowledge of sexual and reproductive health, (b) importance of sexual and reproductive health, (c) sexual and reproductive health problems and their causation, (d) prevention of sexual and reproductive health problems, (e) treatment of sexual and reproductive health problems, and (f) sexual and reproductive health information and sources.

### 3.1. Knowledge of Sexual and Reproductive Health

The study participants had variable understanding of good SRH, and more than half of them identified it as the ability of a person to perform sexual activity and procreate. However, some younger rural men believed that it is the absence of any disease or symptoms in reproductive organs. One of the rural participant said “*Ghar mein jitney jyada baccche matlab baap utna hi swasthya hai*” (the more the number of offsprings, the better the SRH of the father). Two-thirds of participants had correct knowledge about the male reproductive organs and functions especially among urban residents. Most of the rural participants felt the need of better knowledge regarding SRH and were willing to acquire the same. The majority of participants reiterated the deficiency of correct and easily accessible SRH information sources.

### 3.2. Importance of Sexual and Reproductive Health

Most participants felt the importance of SRH and believed that privilege of mobility and freedom of decision making in sexual matters make men more vulnerable and responsible for unfavorable SRH outcomes, for example, STIs/RTIs and unwanted pregnancy. Most rural participants were afraid of such outcomes as they carried social stigma and blame. Nearly all participants agreed that good SRH is important for better fertility outcomes. Around half of the participants said SRH is important matter of concern only for sexually active people and nonindulgence in premarital or high risk sex ensures good SRH. One rural participant said “*Allah ki marzi ke bina sex karne se bimari ho jati hai jisse napunsak ban sakte hain*” (indulging in sex without almighty's wish can lead to disease which can manifest as infertility).

### 3.3. Sexual and Reproductive Health Problems and Their Causation

Around three-fourths of participants had ever heard of one or more SRH problems. It was observed that urban participants enrolled in college had comparatively better knowledge regarding SRH problems, risk factors, and their modes of transmission. Most of the participants felt that male gender role denies them the space to express fears and anxieties regarding personal SRH problems. Participants with higher age were more concerned of sexual performance related issues like sexual weakness or loss of desire (Kamjori, icchanahona), early dysfunction or ejaculation (Jaldi Girna), penile bent (Ling ka tedhapan), infertility or impotency (Napunsakta), and so forth, while younger participants perceived nocturnal emission (Swapnadosh), excess sexual desire (Garmi), and masturbation (Hasthmaithun) as major SRH problems. Most participants felt that SRH problems are caused by highly frequent or risky sexual behavior with some rural participants' quoting “*sex ke side effects*” (side effects of sex).

The majority of participants except few rural residents knew about HIV/AIDS linkage to sexual activity. Urban participants had better knowledge regarding other transmission modes of HIV/AIDS. Awareness regarding other SRH issues, for example, STI/RTI, functional problems, and fertility, was found particularly low among younger rural participants. Frequently mentioned general symptoms of SRH problems were ulcer/sore on private parts, genital discharge, itching around genital areas, burning or pain on urination, and so forth.

Most of the urban and around half of rural participants felt that unhealthy life style (alcohol, tobacco, and drug addiction) and high risk sexual behavior (unprotected sex, visiting CSW, multiple sex partners, etc.) are detrimental to SRH. Few urban participants also mentioned that poor genital hygiene can also increase risk of SRH problems. Knowledge regarding modes of transmission of SRH problems was fair but some rural participants mentioned dirty water, infected blood transfusion, insect bite, and so forth. Rural participants perceived themselves to be at risk of STIs/RTIs due to their lack of SRH knowledge

Most urban participants were able to identify truck/carriage drivers, single men, CSW, widowers, homosexuals, transgender and drug addicts, and so forth as high risk groups for SRH problems. However, rural participants mostly felt that people visiting CSW, indulging in pornography or unnatural sex (homosexuals), and the ones who are not faithful to their partners are at a higher risk of SRH problems. Some rural participants mentioned that sexual activity is meant for procreation and not for recreation quoting “*sex bhagwan ka tohfa hai ek nayi zindagi lane ke liye naaki beja istemaal maze ke liye*” (sex is a gift of God to bring new life to this world not just to misuse it for pleasure) and some rural participants strongly believed that immoral sexual indulgence paves the way to SRH problems.

### 3.4. Prevention of Sexual and Reproductive Health Problems

The majority of participants were aware of the preventive role of condoms in transmission of HIV and AIDS and their contraceptive role but knowledge regarding their preventive role in other STIs was low. Half of the participants felt that being faithful to single partner, avoiding commercial sex workers, and regular medical checkup are important preventive strategies. It was seen that urban participants mostly emphasized role of condoms and proper genital hygiene in prevention of SRH problems while rural participants mentioned reduced high risk behavior. One urban participant said “*jannang ki safai evam nirodh ka istemaal humein aise rogon se bachate hain*” (*genital* hygiene and use of condoms protect us from such diseases). Negative perceptions regarding condom use were widely observed among rural participants. Some rural participants said condom use promotes promiscuous behavior; that is, it is unnatural and not sanctified religiously and hence should be discouraged, while urban participants had different issues regarding condom use and some felt that condoms are difficult to wear and significantly reduce sexual pleasure. Most participants especially rural participants said that condoms are not socially acceptable and it is an embarrassing experience to purchase them. Few participants mentioned the need of premarital SRH screening because of increasing burden of HIV/AIDS. One of the urban participant said “*shadi se pehle yaun rogon ki jaanch jaroor honi chahiye jisse ki dusre ki zindagi barbad na ho”* (it is essential to check for SRH problems before marriage so that health of the other partner can be protected).

### 3.5. Treatment of Sexual and Reproductive Health Problems

Most of the participants said that only few people seek treatment of SRH problems from qualified medical practitioners, primarily due to social stigma and privacy concerns. More than half of participants believed that public health facilities cater only to female SRH problems. While most participants said they had faith and prefer treatment available at public health facilities, some rural participants preferred private doctors, traditional healers (Bengali doctor, vaidyas, hakims, etc.), and home remedies (eating groundnuts, herbs mixed in drinks like milk or buttermilk). Reasons established for alternative treatment seeking behavior were poor knowledge, privacy concern, and callous attitude of doctors and lack of specialists. One rural participant said “*Keval padhe likhe samajhdar log hi sarkari ilaj samajhte hain, baki sabko vaidya ka ilaj chahiye*” (only educated wise people understand treatment at public health facilities and others want treatment by local healers).

### 3.6. Sexual and Reproductive Health Information and Sources

Peer group (friends, colleagues, etc.) and mass media (television, internet, newspaper, books, etc.) were the most important sources for SRH information among rural and urban participants, respectively. SRH information was common regarding HIV/AIDS and condom use as they were the most widely discussed subjects. Peers were the most common sources for SRH information related to personal sexual matters and anxieties. The participants especially the urban felt that SRH information from peer sources might be wrong and could lead to further confusion and increased vulnerability. SRH information received from peers and mass media was most often not fully understood and was frequently ignored by most of the participants. Some rural participants believed that SRH information received through mass media encourages young people to indulge in risky sexual activity. Paucity of correct SRH information was reiterated by participants and some mentioned that SRH chapters in school textbooks were intentionally skipped by teachers. Very few participants received SRH information from health care personnel or dispensary boards or notices in health facilities.

### 3.7. Feedbacks

The majority of participants felt the need for correct and adequate information regarding SRH, especially among urban participants. Information sought was mostly regarding safe ways to have sex, “right time” to start having sex, best practices, prevention of STI, facts regarding sexual physiological issues (night fall, masturbation), and so forth. Half of the participants advocated that SRH is a very important subject and should be formally taught during schooling. Some urban participants said that wide scale dissemination of SRH information will lead to higher societal acceptance and hence better prevention of SRH problems.

## 4. Discussion

Analysis of the FGDs reflects poor SRH knowledge among study participants especially among rural residents. Lack and poor quality of health services were major constraints in adoption of healthy SRH practices. Substantial felt need regarding correct SRH information was observed reflecting lacunae and missed opportunities in public SRH services.

Most participants agreed to the importance of SRH but related it to sexual activity and its outcomes. Importance of SRH was felt but not realized in rural participants mostly due to fear of social stigma, isolation, and blame. Similar findings were observed in rural Nepal where young people rarely visited health posts to seek sexual health services [14]. In our study, SRH problems reported by elder participants were mostly sexual dysfunctions and related anxieties, while younger participants were concerned regarding physiological SRH issues, for example, nocturnal emission, masturbation, and so forth. The majority of participants had good knowledge about HIV/AIDS, but knowledge regarding other STIs/RTIs was poor especially among rural participants. Predictors for better SRH knowledge were higher age, urban residence, postschool education, and so forth among study participants. The findings of our study do not conform to a study conducted at health camp attendees in rural West Bengal, where no statistically significant difference was found in age, monthly income, days of absence from home, or marital status between men reporting SRH problems [11]. Some rural participants perceived themselves to be at higher risk of contracting SRH problems owing to the lack of SRH information. Misconceptions regarding transmission of STIs and immorality ascribed to sexual activity were commonly observed among rural participants similar to the findings of a Nigerian study [15]. The majority of participants held rejective perceptions regarding condom because of difficulty in its use, reduction in sexual pleasure, unnaturality, and religious unacceptance. Most of the participants in our study were doubtful of contraceptive role of condoms and had poor knowledge regarding their use in preventing STIs other than HIV/AIDS. Similar findings were documented in a study among young unmarried men in rural districts of central India where only 68% young men were aware of condoms and their HIV/AIDS preventive role, and 40% participants knew of their role in preventing unwanted pregnancies [5]. In a Nepalese study it was seen that condoms were not easily available in rural markets and their use was often stigmatized; similar findings were seen in the current study [14].

Poor utilization of trained doctors or public health facilities regarding SRH complaints was seen among rural participants due to social stigma, dissatisfaction, callous attitude of staff, lack of specialists, and privacy concerns. Almost half of the participants felt that public health facilities mostly serve women clients in SRH matters. Important SRH knowledge sources were peer group and mass media among rural and urban study participants, respectively. Similar findings were seen in a study conducted in Nepal, where friends and the media, such as newspapers, radios, and television, were the main reported sources of information about sexual matters [14]. However, in our study SRH information received was inadequate and unreliable and mostly related to HIV/AIDS and condom use only.

*Strengths*. Semiqualitative research gives us a broader and in-depth understanding of the sociomedical subject like SRH along with proportional estimates. Inclusion of participants from both rural and urban backgrounds is enabling to understand sociocultural determinants of SRH. It is one of the very few studies conducted to assess young men's perception regarding SRH in North India.

*Limitations*. Data were collected from specified age group of men and may not be representative of the whole range of SRH perceptions among males. The study groups were nonhomogenous in terms of characteristics like age, education, socioeconomic status, and so forth which could have affected the quality of data generated.

## 5. Conclusions

Men have their own SRH concerns and needs which are not always met mostly due to their ascribed gender role and lack of access to specialized health care and information sources. Little knowledge about their own reproductive physiology and SRH problems exposes young men to unsafe behaviors and practices. Young males especially those belonging to rural areas remain disadvantaged in access to correct SRH information compared to urban counterparts, therefore remaining at higher risk of developing SRH problems. Conventional nonformal SRH information sources like peers, mass media, and so forth remain the only source of information to young men and are unreliable and there is a scope of subjective misinterpretations. Providing young men with formal early, correct, comprehensive SRH information can have life-long protective benefits to them along with their partners. IEC activities targeted at this group can prove instrumental in allaying various societal misconceptions about SRH. Peer education programs and increasing access and effective utilization of electronic media especially in rural areas offer potential scope of improvement.

## Abbreviations

FGD: Focus group discussion
SRH: Sexual and reproductive health
STI: Sexually transmitted infection
RTI: Reproductive tract infections
CSW: Commercial sex worker
IEC: Information, education, and counseling.
